# Supplementary material for: A single-cell transcriptomic and anatomic atlas of mouse dorsal raphe Pet1 neurons
Source: eLife. 2020 Jun 22;9:e55523. doi: 10.7554/eLife.55523 (PMC7308082; doi:10.7554/eLife.55523)
Supplement: Figure 8—source data 1. — Table depicting each cluster (column 1) and its proposed anatomical bias (column 2) based on a combination of analysis of histology from intersectional lines and single cell RNA seq (columns 3 and 5), immunostaining and in-situ hybridization (column 4), computational mapping of our subtypes to previously published datasets (column 6), and qualitative analysis of the anatomical distribution of subtype marker genes from the Allen Brain Atlas data set (column 7). As in Figure 8, B7 and B6 here refer to the original Dahlström and Fuxe nomenclature for describing distinct anatomical clusters of 5-HT neurons, and the asterisk after B6 is to indicate that some authors only consider B6 to encompass the dorsal part of what we refer to as the caudal DR. † after ‘Npy2r-cre; Pet1-Flpe rDR scRNAseq and histology’ in row six is to indicate that, while Npy2r-cre; Pet1-Flpe histology shows EGFP positive cell bodies in both dorsal and ventral aspects of the rDR, the scRNAseq data, combined with other evidence given, suggest a more dorsal bias for cluster six DR Pet1 neurons. †† after ‘Slc6a4-cre; Pet1-Flpe vmDR’ in row 11 is to indicate that, while manually sorted Slc6a4-cre; Pet1-Flpe vmDR scRNAseq libraries did not map to cluster 11 overall, cluster 11 neurons nonetheless express several marker genes enriched in the vmDR, which together with other evidence given in the table suggests a vmDR bias. [file elife-55523-fig8-data1.docx]

Figure 8- figure supplement 1

| **Subgroup** | **Proposed Anatomical Bias** | ***Slc17a8-cre; Pet1-Flpe***  **Histology** (Fig 3) | **Immunostaining**  (Fig 3 - supplement 1 and 2,  and Fig 4- supplement 2) | **Manual scRNAseq and Histology** (Fig 3 and Fig 4) | **Previous Publications**  Ren, et al. 2019¹, Huang, et al. 2019² Okaty, et al. 2015³ | **Allen Brain Atlas In-Situ** |
| --- | --- | --- | --- | --- | --- | --- |
| 1 | Rostral (B7) Ventrolateral | *Slc17a8-cre; Pet1-Flpe*  Subtractive Histology |  |  | *Tph2-Npas1* in-situ¹ | *Gabre* and *Gabrq* |
| 2 | Rostral (B7) Dorsolateral and Dorsomedial |  | PAX5 and SATB2 | *Slc6a4-cre; Pet1-Flpe* dlDR and dmDR scRNAseq | *Tph2/Slc6a4-Trh* in-situ¹,² 5-HT-I and II mapping² | *Trh* and *Crym* |
| 3 | Rostral (B7) Dorsomedial |  |  | *Slc6a4-cre; Pet1-Flpe*  dmDR scRNAseq | 5-HT-II mapping² | *Kit* |
| 4 | Rostral (B7) Dorsomedial |  |  |  |  | *Prkcq* |
| 5 | Rostral (B7) Dorsomedial |  | PAX5 |  | *Tph2-Ret* in-situ¹ *Slc6a4-Pdyn* in-situ² 5-HT-III mapping² | *Pdyn* |
| 6 | Rostral (B7) Dorsomedial |  |  | *Npy2r-cre; Pet1-Flpe* rDR scRNAseq and histology† | *Tph2-Ret* in-situ¹ *Tph2-Gad1* in-situ¹ 5-HT-III mapping² | *Amigo2* |
| 7 | Caudal (B6*) Ventromedial | *Slc17a8-cre; Pet1-Flpe*  Intersectional Histology | VGLUT3 and TPH2 NR2F2 | *Npy2r-cre; Pet1-Flpe* cDR scRNAseq and histology |  |  |
| 8 | Caudal (B6*) Ventromedial |  |  | *Slc6a4-cre; Pet1-Flpe* cDR scRNAseq and histology |  | *Rorb* |
| 9 | Rostral (B7) Ventromedial |  |  |  |  | *Nos1* |
| 10 | Rostral (B7) Ventromedial |  | VGLUT3 and TPH2 | *Slc6a4-cre; Pet1-Flpe*  vmDR scRNAseq | 5-HT-IV mapping² |  |
| 11 | Rostral (B7) Ventromedial |  |  | *Rspo3, Foxa1, Proser2 Syt2* expression highest in  *Slc6a4-cre; Pet1-Flpe* vmDR^††^ | *Tph2-Syt2* in-situ¹ | *Foxa1, Tpbg,* and *Pvrl3* |
| 12 | Caudal (B6) Dorsomedial |  | VGLUT3 and TPH2 NR2F2 | *P2ry1-cre; Pet1-Flpe* cDR scRNAseq and histology | *Tph2-Met* in-situ¹ 5-HT-V mapping² MET immuno³ | *Zeb2* |
| 13 | Dorsomedial and Medial |  | VGLUT3 and TPH2  TPH2/*Tph2* immuno/in situ | *Slc6a4-cre; Pet1-Flpe*  subtractive histology |  |  |
| 14 | Distributed |  |  |  |  | *Gpr101* |
