# Supplementary material for: A single-cell transcriptomic and anatomic atlas of mouse dorsal raphe Pet1 neurons
Source: eLife. 2020 Jun 22;9:e55523. doi: 10.7554/eLife.55523 (PMC7308082; doi:10.7554/eLife.55523)
Supplement: Transparent reporting form [file elife-55523-transrepform.pdf]

eLIFE
1st Floor
24 Hills Road
Cambridge CB2 1JP, UK
P 01223 855340
W elifesciences.org
T @elife

eLife's transparent reporting form

We encourage authors to provide detailed information *within their submission* to facilitate the interpretation and replication of experiments. Authors can upload supporting documentation to indicate the use of appropriate reporting guidelines for health-related research (see EQUATOR Network), life science research (see the BioSharing Information Resource), or the ARRIVE guidelines for reporting work involving animal research. Where applicable, authors should refer to any relevant reporting standards documents in this form.

If you have any questions, please consult our Journal Policies and/or contact us: editorial@elifesciences.org.

Sample-size estimation

- You should state whether an appropriate sample size was computed when the study was being designed
- You should state the statistical method of sample size computation and any required assumptions
- If no explicit power analysis was used, you should describe how you decided what sample (replicate) size (number) to use

Please outline where this information can be found within the submission (e.g., sections or figure legends), or explain why this information doesn't apply to your submission:

For single cell RNA-seq experiments, the number of biological replicate animals used (ten mice for 10X scRNA-seq) was consistent with other similar studies reported in the literature. The number of single cells profiled in our study was roughly four-fold higher than other similar studies. This information can be found in the "Single-cell sorting and RNA-sequencing" Methods section and throughout multiple Results sections, including "Droplet-based scRNA-seq of *Pet1* fate-mapped DR neurons reveals new molecularly defined neuron subtypes"**,** **"**Manual scRNA-seq of Pet1-Intersectionally Defined Neuron Populations", and "Comparison to other DR scRNA-seq datasets**".**

Replicates

- You should report how often each experiment was performed
- You should include a definition of biological versus technical replication
- The data obtained should be provided and sufficient information should be provided to indicate the number of independent biological and/or technical replicates
- If you encountered any outliers, you should describe how these were handled
- Criteria for exclusion/inclusion of data should be clearly stated
- High-throughput sequence data should be uploaded before submission, with a private link for reviewers provided (these are available from both GEO and ArrayExpress)

Please outline where this information can be found within the submission (e.g., sections or figure legends), or explain why this information doesn't apply to your submission:

eLife Sciences Publications, Ltd is a limited liability non-profit non-stock corporation incorporated in the State of Delaware, USA, with company number 5030732, and is registered in the UK with company number FC030576 and branch number BR015634 at the address 1st Floor, 24 Hills Road, Cambridge CB2 1JP | August 2014

The number of mice used in studies, the number of cells analyzed, and the number of sample batches is given both in the Results and the Methods for each experiment (e.g. (scRNA-seq, histology, and electrophysiology). For scRNA-seq data, we explicitly detail how data was filtered in the "scRNA-seq analysis" section of the Methods. We are currently in the process of depositing our full RNA-seq dataset to GEO and will provide the accession number once it becomes available.

Statistical reporting

- Statistical analysis methods should be described and justified
- Raw data should be presented in figures whenever informative to do so (typically when N per group is less than 10)
- For each experiment, you should identify the statistical tests used, exact values of N, definitions of center, methods of multiple test correction, and dispersion and precision measures (e.g., mean, median, SD, SEM, confidence intervals; and, for the major substantive results, a measure of effect size (e.g., Pearson's r, Cohen's d)
- Report exact p-values wherever possible alongside the summary statistics and 95% confidence intervals. These should be reported for all key questions and not only when the p-value is less than 0.05.

Please outline where this information can be found within the submission (e.g., sections or figure legends), or explain why this information doesn't apply to your submission:

All statistical tests and the number of animals and cells making up sample groups are explicitly described in the Methods, Results, and in some cases Figure Legends (e.g. Figure 6-figure supplement 1). The specific statistical test employed varied depending on the data modality, and in each case was selected based on consensus in the literature. For example, scRNA-seq data was analyzed using the R package Seurat, which is one of the most widely used approaches to analyzing scRNA-seq data. The full set of differentially expressed genes determined by Wilcoxon Rank Sum tests as implemented in Seurat is given in the Supplemental Excel File, including the magnitude of enrichment and the Bonferroni-corrected p-values.

(For large datasets, or papers with a very large number of statistical tests, you may upload a single table file with tests, Ns, etc., with reference to sections in the manuscript.)

Group allocation

- Indicate how samples were allocated into experimental groups (in the case of clinical studies, please specify allocation to treatment method); if randomization was used, please also state if restricted randomization was applied
- Indicate if masking was used during group allocation, data collection and/or data analysis

Please outline where this information can be found within the submission (e.g., sections or figure legends), or explain why this information doesn't apply to your submission:

All sample groups are unambiguously defined throughout the text, reflecting either mouse genotype (e.g. Figure 3), subgroup definitions based on transcriptomic clustering (with extensive justification given for how these definitions were selected, e.g. Figure 1b,c,d), cell soma anatomy (e.g. as defined in Figure 4a), or subgroups based on neuron firing properties (Figure 6-figure supplement 1).

Additional data files ("source data")

- We encourage you to upload relevant additional data files, such as numerical data that are represented as a graph in a figure, or as a summary table
- Where provided, these should be in the most useful format, and they can be uploaded as "Source data" files linked to a main figure or table
- Include model definition files including the full list of parameters used
- Include code used for data analysis (e.g., R, MatLab)
- Avoid stating that data files are "available upon request"

Please indicate the figures or tables for which source data files have been provided:

The full set of differentially expressed genes is given in the Supplemental Excel File, including the magnitude of enrichment and the Bonferroni-corrected p-values. Also, we are currently in the process of depositing our full RNA-seq dataset to GEO and will provide the accession number once it becomes available.

eLife Sciences Publications, Ltd is a limited liability non-profit non-stock corporation incorporated in the State of Delaware, USA, with company number 5030732, and is registered in the UK with company number FC030576 and branch number BR015634 at the address 1st Floor, 24 Hills Road, Cambridge CB2 1JP | August 2014
